# Variability in Wheelchair Propulsion: A New Window into an Old Problem

**DOI:** 10.3389/fbioe.2015.00105

**Published:** 2015-07-27

**Authors:** Jacob J. Sosnoff, Ian M. Rice, Elizabeth T. Hsiao-Wecksler, Iris M. K. Hsu, Chandrasekaran Jayaraman, Yaejin Moon

**Affiliations:** ^1^Department of Kinesiology and Community Health, University of Illinois at Urbana-Champaign, Urbana, IL, USA; ^2^Department of Mechanical Engineering and Sciences, University of Illinois at Urbana-Champaign, Urbana, IL, USA; ^3^Department of Industrial and Enterprise Systems Engineering, University of Illinois at Urbana-Champaign, Urbana, IL, USA

**Keywords:** motor variability, complexity, wheelchair biomechanics, injuries, kinematics, kinetics

## Abstract

Manual wheelchair users are at great risk for the development of upper extremity injury and pain. Any loss of upper limb function due to pain adversely impacts the independence and mobility of manual wheelchair users. There is growing theoretical and empirical evidence that fluctuations in movement (i.e., motor variability) are related to musculoskeletal pain. This perspectives paper discusses a local review on several investigations examining the association between variability in wheelchair propulsion and shoulder pain in manual wheelchair users. The experimental data reviewed highlights that the variability of wheelchair propulsion is impacted by shoulder pain in manual wheelchair users. We maintain that inclusion of these metrics in future research on wheelchair propulsion and upper limb pain may yield novel data. Several promising avenues for future research based on this collective work are discussed.

There are an estimated 1.5 million manual wheelchair users in the United States (LaPlante and Kaye, 2010). Manual wheelchair users use their upper limbs for mobility and most functional activities. Unfortunately, the human upper limb is not specialized for the repetitive loading required for wheelchair propulsion. This requirement predisposes manual wheelchair users for upper limb pathology. Indeed, up to 70% of manual wheelchair users report upper limb pain (Nichols et al., 1979; Curtis et al., 1999; Gironda et al., 2004), which is mainly manifested in the shoulder and wrist (Dalyan et al., 1999). Furthermore, even in manual wheelchair users who do not report pain, there is evidence of degenerative changes in the shoulder (Lal, 1998), suggesting that it is just a matter of time before these asymptomatic individuals will experience pain.

Upper limb pain in wheelchair users has been linked to difficulty in performing activities of daily living (Dalyan et al., 1999), decreased physical activity, and decreased quality of life (Gutierrez et al., 2007). Overall, any loss of upper limb function due to pain adversely impacts the independence and mobility of manual wheelchair users. It has been speculated that a decrease in independence and mobility results in greater health care costs and an increased risk for secondary morbidity (cardiovascular disease, obesity, etc.) (Silfverskiold and Waters, 1991; Pentland and Twomey, 1994).

The development of upper limb pain in wheelchair users is a multifaceted process (Dyson-Hudson and Kirshblum, 2004). It has been suggested that upper limb pain is related to functional level (Curtis et al., 1999), duration of wheelchair use, wheelchair design (van der Woude et al., 2006), body weight (Sinnott et al., 2000; Collinger et al., 2008), propulsion mechanics (Koontz et al., 2002; Mercer et al., 2006), muscle coordination (Burnham et al., 1993; Kotajarvi et al., 2002), age (Fullerton et al., 2003), and gender (Lal, 1998; Gutierrez et al., 2007). The multi-factorial nature of the possible mechanisms and associated variables creates a daunting task for researchers and clinicians.

## Variability as a Potential Indicator of Upper Extremity Injury

Recently, analysis of motor variability has been utilized as a new approach to understand ergonomic repetitive strain injuries (Srinivasan and Mathiassen, 2012). Although variability measures have been included in investigations focusing on learning of wheelchair propulsion in non-wheelchair users (Vegter et al., 2013, 2014), variability analysis has not been incorporated in investigations of upper extremity pain in manual wheelchair users. To fully understand the potential value of variability analysis to shoulder pain and wheelchair propulsion, it is worthwhile to briefly review this approach.

First and foremost, it is essential to appreciate that variability is inherent within all physiological systems. Despite its ubiquitous status, fluctuations in physiological output including motor variability were historically seen as a nuisance to scientific inquiry; something to be experimentally minimized or altogether eliminated (Newell and Corcos, 1993). However, this approach to variability tends to ignore that variability specifically within an individual can provide important information concerning health and function.

The introduction of non-linear dynamics and chaos theory to motor control and rehabilitation science led to the observation that variability (operationalized as fluctuations of physiological output within an individual) can provide unique information concerning the control and health of the neuromuscular system (Lipsitz, 2004; Sosnoff and Newell, 2006a). Aberrations in health are frequently denoted by a change in within individual variability (Sosnoff and Newell, 2006b). Examining variability in health has led to important insights in understanding the development of overuse injuries. Optimal musculoskeletal health results from repetitive sub-maximal loading with a certain amount of variability in frequency (i.e., timing) and rate of loading (i.e., force application) (Hamill et al., 1999). It is maintained that a lack of variation results in insufficient time to adapt (i.e., heal) between loading occasions. To date, a relation between kinematic variability and skeletal injury has been demonstrated in individuals with knee (Hamill et al., 1999), shoulder (Madeleine et al., 2008), and low-back pain (Lamoth et al., 2006).

For instance, a series of investigations examining upper limb occupational tasks, such as butchering, have reported an increase in arm movement variability in individuals with musculoskeletal pain (Madeleine et al., 2008; Lomond and Cote, 2011). Additionally, studies examining repetitive reaching tasks demonstrate that subjects with shoulder pain exhibited higher relative variability in their kinematics than those without pain (Lomond and Cote, 2010, 2011). Based on this collective body of work, we have speculated that variability in wheelchair propulsion is related to shoulder pain in manual wheelchair users. The purpose of this local review is to discuss published and unpublished research examining variability in wheelchair propulsion as a function of shoulder pain from our research group.

## Variability and Wheelchair Propulsion: Recent Investigations

Recently, our research group at the University of Illinois at Urbana-Champaign supported by the National Institute of Health (#1R21HD066129-01A1) has set out to apply variability analyses to wheelchair propulsion. Specifically, we have conducted several investigations examining the association between variability in wheelchair propulsion and shoulder pain in manual wheelchair users.

### Experimental set up

The data incorporated into these investigations (Moon et al., 2013; Jayaraman et al., 2014; Rice et al., 2014a) were derived from the same experimental set up. For brevity, the experimental setup and methodology will be described prior to detailing the actual investigations. Specifically, experienced manual wheelchair users with a range of physical disabilities propelled their own wheelchairs that where equipped with force sensing wheels (Smartwheels™) at a steady state pace on a dynamometer at three different speeds (self-selected, 0.7 m/s, 1.1 m/s) for 3 min. The use of force sensing wheels allowed for the determination of temporal–spatial and kinetic data relating to wheelchair propulsion. Additionally, we collected kinematic data on arm motion using a 10 camera motion capture system (Raptor Digital RealTime System, Motion Analysis Co., Santa Rosa, CA, USA), which tracked reflective markers on the participant’s upper body bony landmarks. Based on international society of biomechanics recommendations (Wu et al., 2005), 18 reflective markers were attached bilaterally, at specific bony landmarks on the following locations: third metacarpophalangeal joint (i.e., middle finger knuckle), radial styloid (outside of writs), ulnar styloid (inside of wrist), olecronon process (tip of elbow), lateral epicondyl, acromion (front of shoulder), sternal notch (chest), C7 vertebrae (base of neck), T3 vertebrae (base of skull), T6 vertebrae (middle region of the spine), and jaw.

### Wheelchair propulsion variability: Experimental data

**Figure 1:**
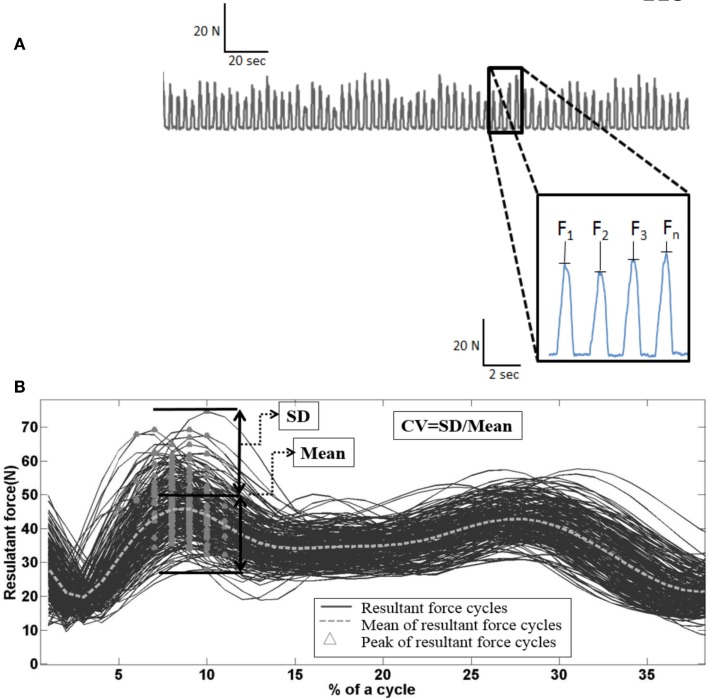
**(A)** Peak hand-rim resultant force profile as a function of time during steady state wheelchair propulsion. Inset illustrates subtle variations in peak force over four pushes. **(B)** Resultant shoulder force output during the push phase of ~300 pushes of steady state wheelchair propulsion. Dashed line depicts mean resultant force, while triangles depict individual cycle peak resultant shoulder force.

Figure 1A depicts the resultant force profile over 2 min of wheelchair propulsion of an individual with spinal cord injury. Subtle variations in the force profile between individual pushes are evident. Traditionally, researchers have averaged across the force profile of individual push cycles. Our first investigation sought to determine whether intra-individual variability of kinetic and temporal–spatial parameters of wheelchair propulsion was distinct in manual wheelchair users with and without shoulder pain (Rice et al., 2014a).

In this investigation, data from 26 adults [with shoulder pain (*n* = 13) and without shoulder pain (n = 13)] with a range of physical disabilities who use a manual wheelchair for mobility were analyzed. Specifically, intra-individual mean, SD, and coefficient of variation of (CV = mean/SD) of kinetic and temporal–spatial metrics were determined for salient spatiotemporal events (e.g., push time, peak push force, etc.).

Consistent with previous research (Mercer et al., 2006; Collinger et al., 2008), shoulder pain had no influence on mean kinetic and temporal–spatial propulsion variables at the hand-rim. However, significant group differences were found in relative variability (i.e., CV). Specifically, individuals with shoulder pain displayed less relative variability in their cycle-to-cycle peak resultant force and push time than individuals without shoulder pain. These preliminary results suggest that intra-individual variability analysis is sensitive to shoulder pain.

In a subsequent investigation, our research team examined the variability of peak resultant force acting on the shoulder during the push phase of wheelchair propulsion in individuals with and without self-reported shoulder pain (Moon et al., 2013). Figure 1B illustrates resultant force acting on the shoulder of a participant during steady state wheelchair propulsion. It is apparent in the figure that there are significant fluctuations in peak force from cycle to cycle. Propulsion data from 24 manual wheelchair users (13 with pain, 11 without pain) were included in the investigation. Peak resultant shoulder forces in the push phase were calculated using inverse dynamics. Mean, SD, and coefficient of variation of cycle-to-cycle peak resultant forces were calculated and analyzed as a function of shoulder pain.

Consistent with previous reports (Mercer et al., 2006; Collinger et al., 2008), we found no difference in mean peak shoulder resultant force between pain groups [no pain (41.38 ± 3.06 N) versus pain (44.16 ± 3.06 N)]. However, the pain group had significantly smaller variability of peak resultant force than the no pain group. These observations further raise the possibility that variability during the push phase of wheelchair propulsion maybe related to upper limb pain in manual wheelchair users.

In another investigation, we focused on intra-individual variability during the recovery phase of wheelchair propulsion as a function of shoulder pain (Jayaraman et al., 2014). Given that the recovery stroke is dependent upon the propulsion pattern employed (Sanderson and Sommer, 1985; Shimada et al., 1998), this investigation only included individuals who utilized a semi-circular propulsion pattern. Specifically, data from 10 experienced adult manual wheelchair users with spinal cord injury (5 with shoulder pain; 5 without shoulder pain) were analyzed. Intra-individual kinematic spatial variability of the steady state wrist motion during the recovery phase was determined using principal component analysis (PCA). PCA belongs to the factor analysis family and is a statistical decomposition technique used to identify patterns in data, thus, highlighting data similarities and differences (Daffertshofer et al., 2004).

**Figure 2:**
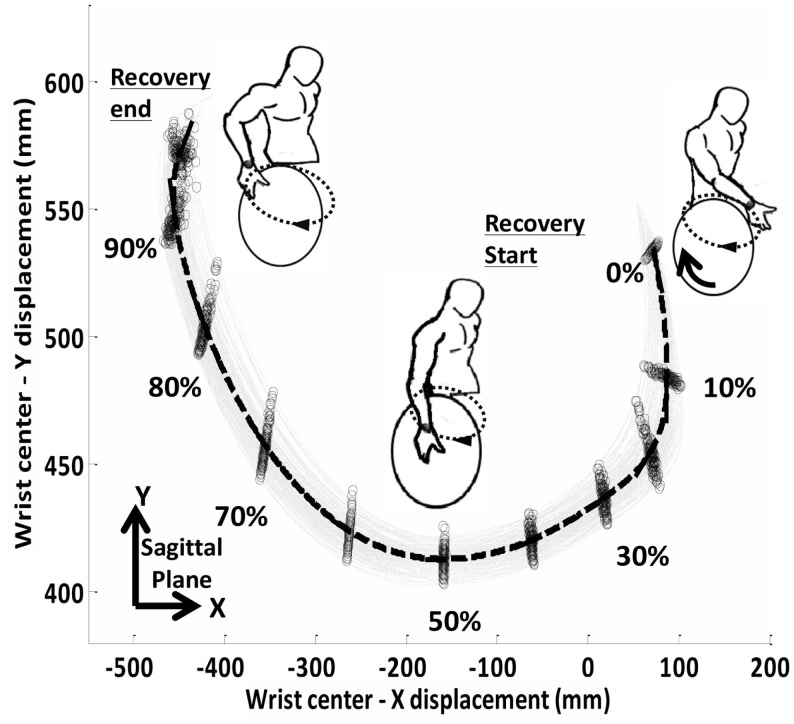
**Wrist recovery trajectories during semi-circular pattern wheelchair propulsion**. Wrist cycle-to-cycle recovery trajectories (“gray solid lines”). The mean wrist recovery trajectory is shown by the bold dashed line. The wrist positions orthogonal to mean recovery trajectory for which PCA was computed (0–100% at every 10% interval along the recovery path) is denoted by (“o”).

Utilizing this technique, the kinematic spatial variability was calculated at 10% intervals along the wrist recovery path. Spatial variability was found to be highest at the start and end of the recovery path and lowest during the middle of the recovery path (Figure 2). Additionally, individuals with shoulder pain displayed significantly higher kinematic spatial variability than individuals without shoulder pain at the start (at 10% interval) of the recovery phase.

This pilot investigation further highlights that the analysis of intra-individual variability during manual wheelchair propulsion can distinguish between those with and without shoulder pain. It provides further evidence that variability analysis of wheelchair propulsion may offer a new approach to examine the impact of shoulder pain.

It is important to note that the association between pain and variability was distinct between the investigations that focused on push and recovery phase of wheelchair propulsion. Indeed, the first two investigations (Moon et al., 2013; Rice et al., 2014a) reported that those with shoulder pain had less variability than those without out; however, the investigation that exclusively focused on recovery phase demonstrated that those with pain had greater variability in their movement. There are several potential explanations for this discrepancy. Perhaps, the most straightforward is the difference in kinetics versus kinematics. It is possible that participants constrained their movement when applying pressure to the hand-rim in an effort to stay in a “pain free/minimization” zone. However, when their arm is unconstrained, they are more variable. Indeed, research focusing on unconstrained reaching tasks has demonstrated that those with shoulder injury/pain have greater kinematic variability than those without pain (Lomond and Cote, 2010, 2011). It is important to note that, Hamill et al. (2012) have theorized that musculoskeletal injury, such as shoulder pain in manual wheelchair users, can develop from either too little or too much motor variability. The complex relationship between motor variability and musculoskeletal injury warrants further investigation.

The collective findings also highlight that the importance of identifying the appropriate wheelchair propulsion variable to investigate. The variables that we have examined were based on previous reports (Morrow et al., 2009) and accepted practice in the field. It is quite possible that variability of other measures is more informative. For instance, it has been suggested that the variability of the interaction between segments or joints (i.e., coordinative variability) plays a key role in patella-femoral pain syndrome (Hamill et al., 2012). Further work is necessary to determine the appropriate variables of study.

## Novel Approaches to Examine Variability in Wheelchair Propulsion

In addition to the published investigations detailed above, we have also conducted several preliminary analyses focusing on novel variability metrics. For instance, recently, we have sought to determine whether temporal variations between strokes are random or rather have some quantifiable structure, such as walking (Hausdorff, 2007). In this preliminary investigation data from 13 experienced adult manual wheelchair users with spinal cord injury were analyzed. A time series of resultant force at hand-rim was computed from the raw SMARTWheel data. To maintain consistency on the number of data points analyzed across individuals, only data from 100 cycles from each participant were used. Based on the occurrence of peak resultant force event on each cycle, two measures were extracted, namely, (1) a time series of cycle peak resultant force amplitude (PFR) and (2) a time series of inter-push time interval between peak resultant force (IPT) (Figure S1 in Supplementary Material). To investigate if the temporal variability observed in peak resultant force and inter-push time were random or had time-dependent structure, 1000 randomly shuffled surrogate time series were produced from each original time series. Each surrogate time series has the same distributional properties (mean and variance) as its corresponding original time series except that the order of occurrence of data points is randomized. Following the generation of surrogate time series, sample entropy (SampEn) of the original and each of its surrogate time series were computed. SampEn, is a metric that quantifies the regularity of a time series (Yentes et al., 2013). The SampEn of each original time series was then compared to the mean SampEn of surrogated counter parts (Paired *t*-test, two-tailed, α = 0.05).

As expected, the original and surrogated data had identical mean (SD) of peak resultant force and inter-push time as 57.21 (16.63) N and 1.15 (0.22) s, respectively. Statistical analysis revealed that the SampEn of the original time series was significantly different than the surrogated time series for both peak resultant force and inter-push interval (*p*’s < 0.05). The mean sample entropy for the surrogate time series [PFR: 2.13 (0.12); IPT: 2.02 (0.26)] was higher than that obtained from the original time series [PFR: 2.07 (0.13); IPT: 1.87 (0.25)]. These preliminary results indicate that time- and amplitude-dependent variability in resultant force observed in wheelchair propulsion are not random and have quantifiable structure. A significant limitation of this pilot investigation is that the time series of propulsion data is relatively small (*n* = 100 data points) for this type of analysis. It remains to be determined whether or not this structure is informative of upper extremity injury or other adverse consequences of wheelchair propulsion.

In another analytical approach, we examined the variability of arm motion during wheelchair propulsion utilizing phase portraits (Hsu et al., 2012). Phase portraits, which are graphical representations of position relative to velocity, can be used to explore the dynamics of a system over multiple cycles. We implement techniques developed to examine changes in variability and complexity in the shape of phase portraits. Variability was quantified by examining fluctuations of the centroid of each phase portrait over multiple cycles, specifically by calculating the confidence area and drift of the centroid. Complexity of the portrait was quantified by determining the portrait shape’s frequency content using Fourier-based methods (DiBerardino et al., 2010). In this preliminary analysis, phase portraits of shoulder flexion–extension angular position versus angular velocity were examined as function of propulsion speed (see Figure S2 in Supplementary Material).

Data from nine experienced manual wheelchair users were analyzed in this pilot analysis. Variability parameters had mixed results with propulsion speed. There was a trend for the centroid area to increase with speed; whereas there was no significant change in centroid drift. Complexity of the phase portrait shape decreased significantly with speed. These results support prior work that propulsion speed impacts shoulder biomechanics (McGregor et al., 2009). Future work needs to determine if variability and complexity metrics of phase portrait are sensitive to shoulder pain similar to other metrics that we have utilized.

## Limitations

Despite the novelty of this body of research, it was not without limitations. Specifically, these investigations included individuals who were manual wheelchair users, regardless of disability. Consequently, it is possible that differences in propulsion variability between pain groups was due to different disability being represented in each group and not shoulder pain *per se*. We do note that removal of participants without spinal dysfunction did not change the observe results in any of the reported studies and that ~80% of the sample were individuals with spinal dysfunction. The data were collected on a roller dynamometer, so it is not clear if these differences in propulsion variability would occur in over ground propulsion. Additionally, the use dynamometer precludes examination of some viable metrics, such as left-right coupling of steering (Vegter et al., 2013). It is also important to note that these investigations, classified individuals based on self-report of shoulder pain and no radiological information was collected. Future research utilize other measures of upper extremity pain are warranted. The association between pain in other joints, such as the wrist and elbow, and propulsion variability is not clear. Perhaps, the largest limitation is that this research is cross-sectional in nature, so no conclusions regarding causation can be made. We note that although these are significant limitations they are relatively common to wheelchair propulsion research.

## Future Steps in Variability Analysis in Wheelchair Propulsion

The reviewed work highlights that variability of wheelchair propulsion maybe related to shoulder pain. We maintain that these metrics should be included in future research on wheelchair propulsion and upper limb pain. There are several promising avenues for future research based on this collective work.

The most important and perhaps most difficult step is to examine whether within individual variability in wheelchair propulsion is predictive of development of shoulder pain. We note that the majority of investigations that have attempted to determine predictors of shoulder pain in manual wheelchair users have been inconclusive (Mercer et al., 2006; Collinger et al., 2008).

It would also be worthwhile to examine whether training can alter wheelchair propulsion variability. Propulsion training is often viewed as a low-cost high-impact rehabilitation approach in upper limb preservation in manual wheelchair users (Boninger et al., 2005; de Groot et al., 2008; Rice et al., 2014b). Although variability of wheelchair propulsion has been examined in novice wheelchairs users as a function of training (de Groot et al., 2003), it is not clear if there would be changes in propulsion variability in experienced users with targeted training.

Just as variability analyses have provided insight into musculoskeletal injury in the ambulatory population (Hamill et al., 2012; Srinivasan and Mathiassen, 2012), we maintain that this approach has much promise in wheelchair users. Despite this promise of this theoretical view, there is much research to be done. We maintain that these series of investigation are a move in the right direction to understanding upper extremity pain in manual wheelchair users.

## Conflict of Interest Statement

The authors declare that the research was conducted in the absence of any commercial or financial relationships that could be construed as a potential conflict of interest.

## Supplementary Material

The Supplementary Material for this article can be found online at http://journal.frontiersin.org/article/10.3389/fbioe.2015.00105
