# Supplementary material for: Variability in Wheelchair Propulsion: A New Window into an Old Problem
Source: Front Bioeng Biotechnol. 2015 Jul 27;3:105. doi: 10.3389/fbioe.2015.00105 (PMC4515595; doi:10.3389/fbioe.2015.00105)

Supplementary Figures

Figure S1 | Schematic of calculation of inter-push interval and peak force during steady-state wheelchair propulsion for complexity analysis

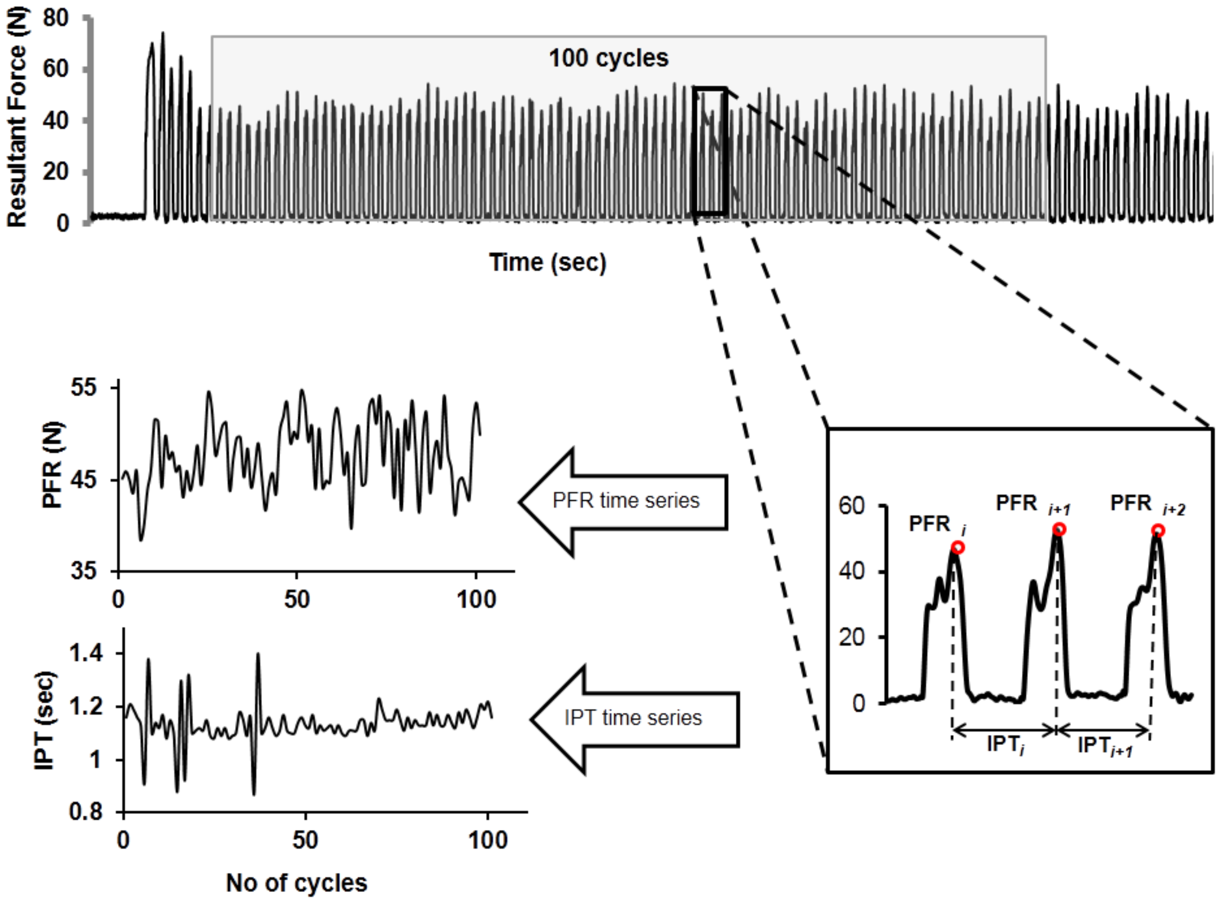

Supplement: Supplementary file 1 [file Image_1.PDF]
