# Supplementary material for: Variability in Wheelchair Propulsion: A New Window into an Old Problem
Source: Front Bioeng Biotechnol. 2015 Jul 27;3:105. doi: 10.3389/fbioe.2015.00105 (PMC4515595; doi:10.3389/fbioe.2015.00105)
Supplement: Supplementary file 2 [file Image_2.PDF]

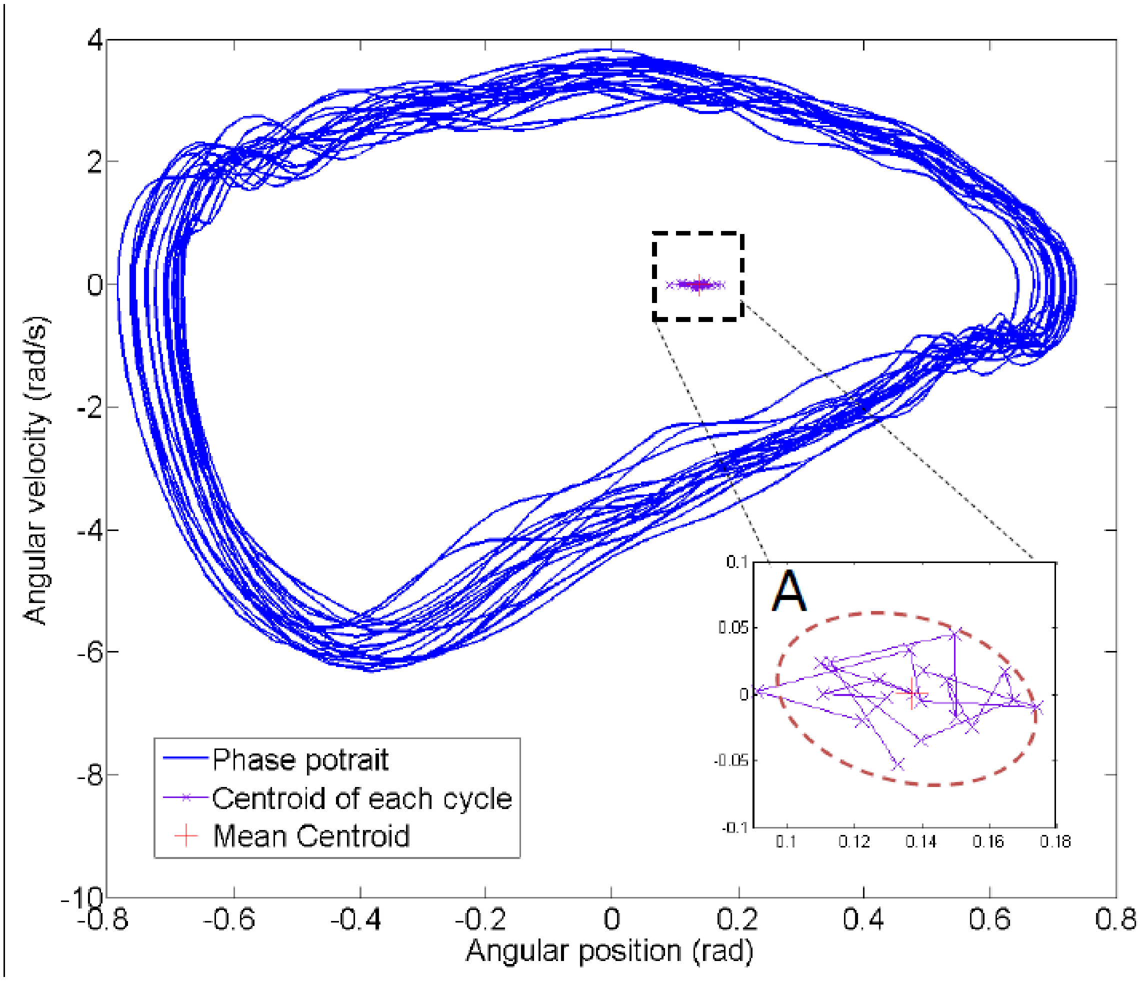

Figure S2 | . Phase portrait of upper arm motion during steady state wheelchair propulsion. Inset depicts centroid variability as a function of cycle.
